# Cordycepin: a dual-function molecular element for aptamer engineering with enhanced anticancer activity†

**DOI:** 10.1039/d5sc02571k

**Published:** 2025-06-30

**Authors:** Fei Gao, Li Na, Shuyue Fu, Jinsong Peng, Shipeng He, Ruowen Wang, Weihong Tan

**Affiliations:** a Institute of Translation Medicine, School of Life Science, Shanghai University Shanghai 200444 China heshipeng@shu.edu.cn; b Institute of Molecular Medicine (IMM), Renji Hospital, State Key Laboratory of Systems Medicine for Cancer, Shanghai Jiao Tong University School of Medicine, College of Chemistry and Chemical Engineering, Shanghai Jiao Tong University Shanghai 200240 China wangwrw@sjtu.edu.cn tan@him.cas.cn

## Abstract

Cordycepin (3′-deoxyadenosine, 3′-dA), derived from the fungus *Cordyceps sinensis*, has shown significant bioactivity as an inhibitor of enzymes related to 2′-deoxyadenosine (dA). However, its therapeutic efficacy is insufficient for clinical use, which may be addressed through targeted delivery systems. In this study, we designed and synthesized a 3′-dA phosphoramidite to incorporate cordycepin into the well-known cancer-targeting Sgc8c aptamer, where it functions both as a structural modulator and as a bioactive drug element for constructing aptamer–drug conjugates. Its structural similarity to dA makes cordycepin a unique molecular tool for probing the structure–activity relationship of aptamers. Additionally, cordycepin can be seamlessly integrated into aptamers, replacing dA. This led to the generation of a series of cordycepin-modified aptamers, among which Sgc8-23A demonstrated enhanced antitumor activity against HCT116 human colon cancer cells. Compared to free cordycepin, Sgc8-23A exhibited superior bioactivity and stability. In a zebrafish patient-derived xenograft (PDX) model, Sgc8-23A significantly inhibited tumor growth, highlighting its potential as an effective aptamer–drug conjugate for targeted cancer therapy. These findings emphasize the dual functional potential of cordycepin as both a structural element for aptamer optimization and a therapeutic drug component, paving the way for the development of more efficient aptamer-based drug delivery systems.

## Abstract 2

This study demonstrates that cordycepin (3′-dA), as a dual-function element, enhances the Sgc8c aptamer's antitumor activity and stability. Sgc8-23A shows improved efficacy in colon cancer models, highlighting its potential for targeted therapy.

## Graphical abstract

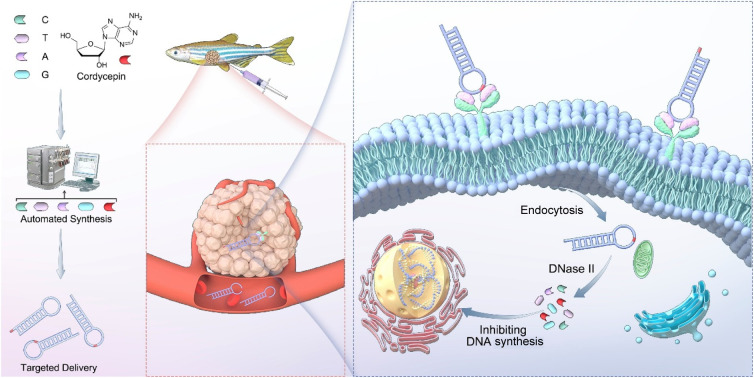

## Introduction

Cordycepin, a naturally occurring nucleoside derived from the fungus *Cordyceps sinensis*, has been a staple of traditional Chinese medicine for centuries.^1,2^ Recent studies have highlighted the therapeutic potential of 3′-dA across multiple biological targets.^3^ Notably, a derivative of 3′-dA has been developed as its prodrug and is currently undergoing phase I clinical trials.^4,5^ Despite their promising attributes, the relatively mild efficacy of natural products such as 3′-dA can often be optimized through targeted delivery systems. Previous work has demonstrated that aptamers serve as an effective tool for achieving such targeted delivery.^6,7^

Aptamers are functional nucleic acids with intricate secondary and tertiary structures capable of binding a wide range of targets, including small molecules, proteins, and even whole cells, with remarkable affinity and specificity.^8,9^ Over the past few years, numerous aptamers have been explored for diagnostic and therapeutic applications, addressing conditions such as Alzheimer's disease and cancer.^10,11^ Owing to their synthesis *via* automated solid-phase methods, aptamers are often referred to as “chemical antibodies”. Aptamers, selected through SELEX (Systematic Evolution of Ligands by Exponential Enrichment) technology, do not always require chemical modifications, but such modifications can be beneficial for overcoming *in vivo* challenges associated with oligonucleotide therapeutics. For example, the FDA-approved aptamer-based therapeutics Pegaptanib for treating wet age-related macular degeneration, or wet AMD, and avacincaptad pegol (brand name: Izervay) for treating geographic atrophy secondary to AMD both incorporate polyethylene glycol (PEG) modifications to enhance biostability and prolong circulation times.^12,13^ Structural optimization of lead compounds, guided by the crystal structures of target proteins, is a common strategy in drug discovery and has led to the development of “perfect molecules” for therapeutic purposes. However, obtaining detailed structural information on oligonucleotides poses significant challenges. Recent advances in structural biology have begun to address these limitations, facilitating the study of aptamer structures.^14^

Cordycepin, a 3′-dA structurally similar to the DNA nucleoside dA, introduces subtle, yet impactful, differences when substituted into aptamer sequences.^3,15^ Thus, this modification may serve as an invaluable model within the field of nucleic acids for improving the antitumor ability and stability of aptamers. Such replacements lead to analogs with delicate changes in secondary structures and distinct binding affinities.^16–19^ This unique property makes 3′-dA a valuable dual-function molecular element for aptamer optimization, enabling the exploration of aptamer structure–activity relationships. Additionally, cordycepin-modified aptamers offer dual functionality. That is, the targeted delivery of aptamers ensures cellular internalization, while the subsequent release of cordycepin provides therapeutic benefits. This dual-function nature positions cordycepin as a powerful tool for both aptamer structural refinement and therapeutic application.

**Fig. 1:**
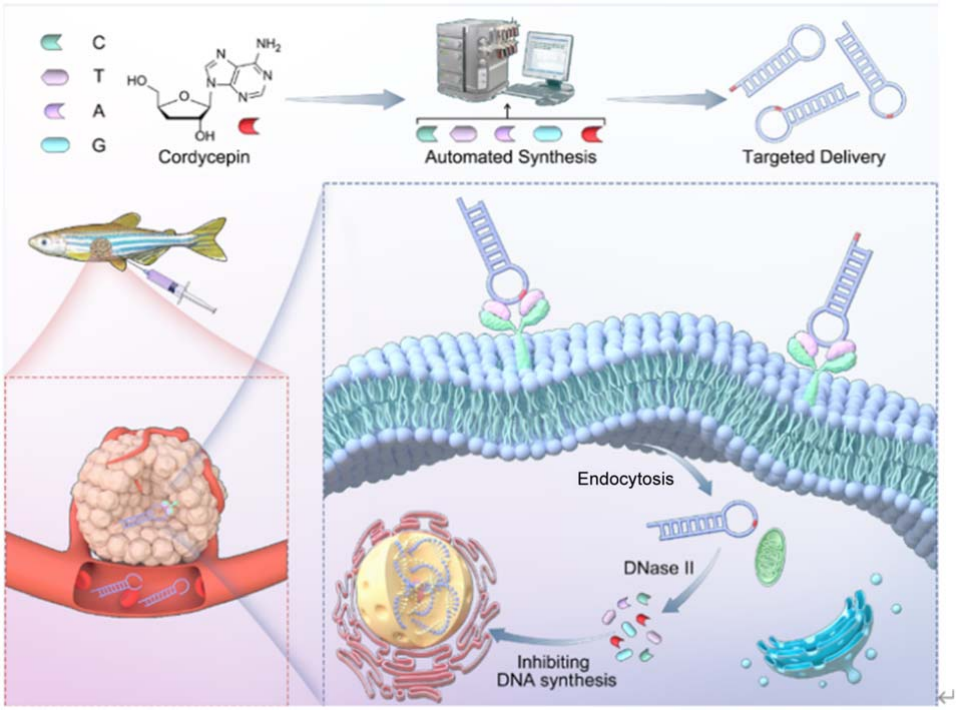
The design of aptamers with cordycepin as a dual-function aptamer element.

Given this duality, we propose a novel strategy to design cordycepin as a dual-function molecular element and incorporate it into the nucleotide sequence of aptamer Sgc8c*via* solid-phase synthesis to replace deoxyadenosine (Fig. 1). This prodrug-like approach aims to enhance the bioavailability and therapeutic efficacy of aptamer Sgc8c. By substituting adenine nucleotides with cordycepin, we created a series of modified Sgc8c aptamers, allowing us to investigate the resulting changes in their properties. Remarkably, cordycepin-modified Sgc8-23A not only retained its superior targeting ability towards HCT116 human colon cancer cells but also exhibited significantly enhanced stability and antitumor activity. The advantages of the cordycepin-modified Sgc8c (Sgc8-23A) were further highlighted by its excellent *in vivo* tumor-targeting ability and potent antitumor efficacy. Therefore, the cordycepin-modified Sgc8c strategy may offer a valuable tool for the clinical diagnosis and targeted treatment of tumors by enhancing the antitumor efficacy and stability of Sgc8c.

## Results and discussion

### Rational design of compounds

Cordycepin (3′-dA), a nucleoside antibiotic structurally similar to adenosine, has been under consideration as an anticancer drug based on its extensive biological and pharmacological effects, which include inhibiting cell proliferation, inducing apoptosis, preventing metastasis, and activating the immune system.^20–22^ Research suggests that 3′-dA is phosphorylated by certain phosphate kinases to 3′-deoxyadenosine triphosphate (3′-dATP) after entering cells.^23^ Owing to its structural resemblance to ATP, 3′-dATP can terminate RNA elongation by incorporating into RNA and may compete with ATP for the EGFR binding site, thereby inhibiting EGFR phosphorylation and disrupting caspase and GSK-3β pathways.^15,24–26^ Additionally, 3′-dA has been suggested to mediate apoptotic signaling in tumor cells through death receptors (DRs) or adenosine receptors (ADORAs).^27^ However, the efficacy of 3′-dA was limited by its rapid metabolism into an inactive metabolite by adenosine deaminase (ADA). Therefore, given the chemical similarity between cordycepin and deoxyadenosine, substituting deoxyadenosine with cordycepin in the Sgc8c aptamer is anticipated to enhance cordycepin's bioavailability. Furthermore, research substantiated that 2′–5′ phosphodiester bonds demonstrated significantly enhanced resistance to nuclease degradation compared to the traditional 3′–5′ bonds. Thus, this modification may serve as an invaluable model within the field of nucleic acids for improving the antitumor ability and stability of aptamer Sgc8c.

**Scheme 1:**
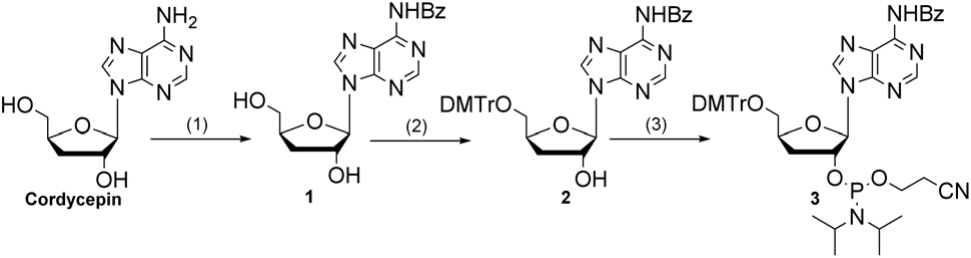
The synthesis of cordycepin phosphoramidite. (1) TMSCl, pyridine, BzCl, NaHCO_3_, 0 °C, 41%; (2) DMTrCl, pyridine, 51%; (3) chlorophosphoramidite, DIEA, DCM, 91%.

The synthesis of aromatic polycarbonates (aPCs) is outlined in Scheme 1. The pharmaceutical element, cordycepin phosphoramidite 3, was prepared in three steps starting from commercially available cordycepin. First, trimethylsilyl chloride (TMSCl) was employed to protect the free amine, affording the intermediate 1 with a yield of 41%. This protection step is essential in solid-phase synthesis to prevent undesired side reactions. Intermediate 1 was then converted to the corresponding phosphoramidite 3 using standard protocols. With the phosphoramidite 3 in hand, we proceeded to synthesize cordycepin-modified Sgc8c to explore how the incorporation of cordycepin affects the overall properties of the aptamer. Based on previous studies,^7^ the A23-T30 region and the terminal sequences of Sgc8c are modifiable sites, and modifications at these positions still preserve the Sgc8c's strong binding affinity. As a result, a series of cordycepin-modified aptamers were successfully prepared (Table S1†).

### Structural integrity and stability of cordycepin-modified Sgc8c

**Fig. 2:**
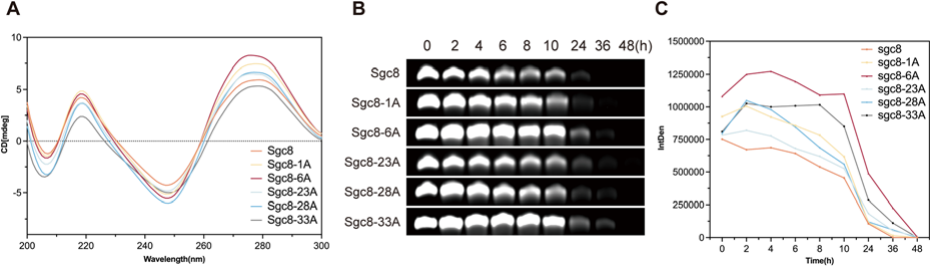
Functional characterization of cordycepin-modified Sgc8c. (A) The secondary structure of Sgc8c and its cordycepin-modified aptamer derivatives was analyzed using circular dichroism. (B) The biostability of cordycepin-modified aptamers was evaluated using PAGE analysis after incubation in RPMI-1640 medium with 10% FBS.

Subsequently, we evaluated the secondary structure and stability of cordycepin-modified Sgc8c. As reported in the literature, replacing the 3′–5′ phosphodiester bond linkage in oligonucleotides with a 2′–5′ linkage can lead to structural alterations.^28,29^ To investigate these effects, we examined the secondary structure of cordycepin-modified Sgc8c using circular dichroism (CD) spectroscopy. As shown in Fig. 2A, cordycepin-modified Sgc8c, similar to aptamer Sgc8c, displays distinct absorption peaks at 255 nm and 280 nm. Among the different modifications, Sgc8-23A exhibited the smallest change in absorbance, indicating minimal structural alteration, while Sgc8-6A showed the largest change, suggesting the most significant structural modification. These results demonstrated that modifications at different sites had varying effects on the properties of aptamer Sgc8c. Subsequently, nuclease resistance of the cordycepin-modified aptamers was assessed *via* PAGE analysis after incubation in RPMI-1640 medium supplemented with 10% fetal bovine serum (FBS) at 37 °C. The results indicated that the cordycepin-modified aptamers demonstrated greater stability in biological environments compared to aptamer Sgc8c (Fig. 2B).

### Binding affinity and cellular internalization of cordycepin-modified Sgc8c

**Fig. 3:**
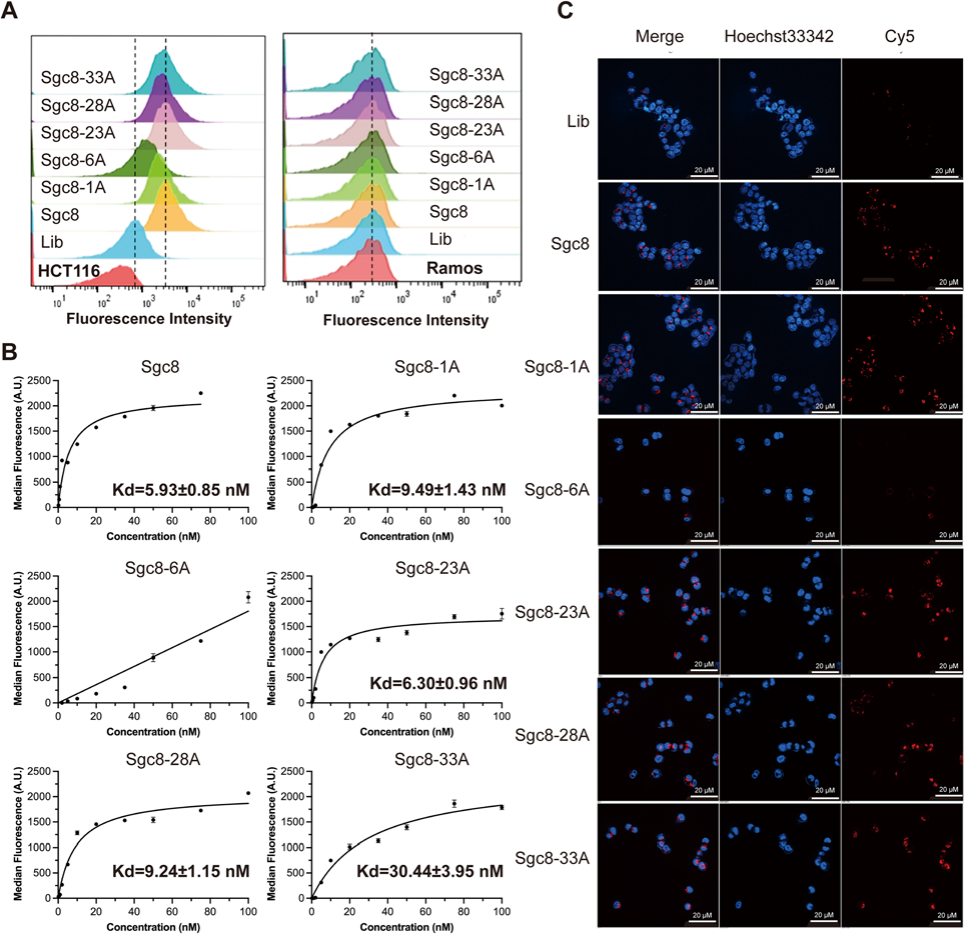
Specific recognition and internalization of cordycepin-modified Sgc8c on HCT116 cells. (A) Flow cytometric results indicating the specific binding of cordycepin-modified Sgc8c to target HCT116 cells. All aptamers were labeled with Cy5 at the 3′ ends (Cy5 excitation at 650 nm and emission at 670 nm). Cells were incubated with DNA at a concentration of 250 nM in binding buffer for 30 min at 4 °C. (B) Dissociation constant of cordycepin-modified Sgc8c in HCT116 cells (*n* = 3). (C) Confocal fluorescence microscopy images of HCT116 cells treated with 500 nM Cy5-labeled (red) cordycepin-modified Sgc8c binding buffer at 37 °C for 2 h. The nucleus was counterstained with Hoechst 33342 (blue); scale bar, 20 μm.

**Fig. 4:**
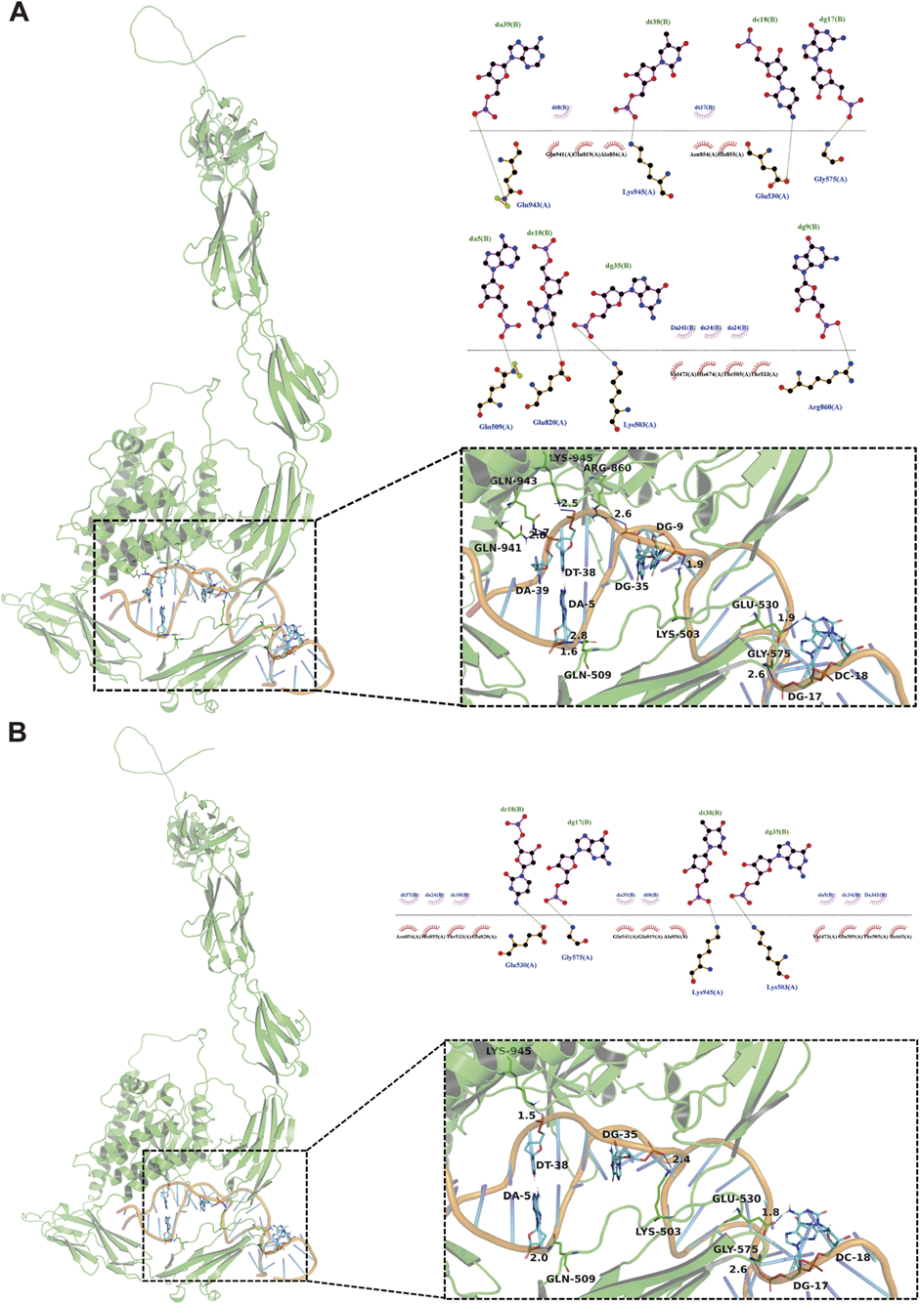
Molecular docking. (A) Molecular docking of Sgc8c with the PTK 7 protein. (B) Molecular docking of Sgc8-23A with the PTK 7 protein.

Aptamer Sgc8c is well known for its specific recognition of the PTK 7 receptor overexpressed in various types of tumors.^30^ Studies indicate that the aptamer's binding affinity is closely related to its structural stability because increased binding affinity is correlated with increased stability.^31^ To explore how cordycepin substitution at different positions affects the binding and internalization abilities of Sgc8c, we used flow cytometry to assess changes in binding specificity and laser confocal microscopy to evaluate internalization efficiency. As shown in Fig. 3A, the fluorescence of HCT116 cells incubated with Sgc8-1A, Sgc8-23A, Sgc8-28A, or Sgc8-33A was comparable to that of Sgc8c and significantly higher than that of the negative control group (Lib, a random sequence). Notably, Sgc8-6A led to weaker fluorescence. In Ramos cells, cordycepin-modified aptamers showed fluorescence levels as low as those in the Lib group. The results demonstrated that these modified aptamers maintain exceptional specificity for tumor cells. To gain deeper insight into the changes in binding affinity between cordycepin-modified aptamers and the receptor, we also used flow cytometry to measure the *K*_d_ value of the nucleic acid aptamers. The *K*_d_ value of Sgc8-23A (6.30 ± 0.95 nM) was closest to that of Sgc8c (5.92 ± 0.84 nM), while Sgc8-6A displayed a nonspecific linear trend, indicating a lack of specific binding to HCT116 cells (Fig. 3B). Confocal microscopy was employed to assess cellular internalization of the cordycepin-modified aptamers. As displayed in Fig. 3C, HCT116 cells treated with Sgc8-1A, Sgc8-23A, Sgc8-28A, or Sgc8-33A displayed significantly stronger fluorescence compared to the Lib group. In contrast, the fluorescence of HCT116 cells incubated with Sgc8-6A was comparable to that of the Lib sequence, further suggesting that Sgc8-6A lacks substantial specific binding affinity for HCT116 cells. Furthermore, molecular docking analyses were performed to explore the binding interactions of both Sgc8c and Sgc8-23A with proteins. As shown in Fig. 4, the predicted binding mode of Sgc8-23A (Fig. 4B) closely mirrors the orientation observed for Sgc8c (Fig. 4A). This similarity in binding patterns between the parent aptamer and its modified variant provides further support for the potential functional equivalence of Sgc8-23A.

### Tumor inhibitory activity of cordycepin-modified Sgc8c on HCT116 cells

**Fig. 5:**
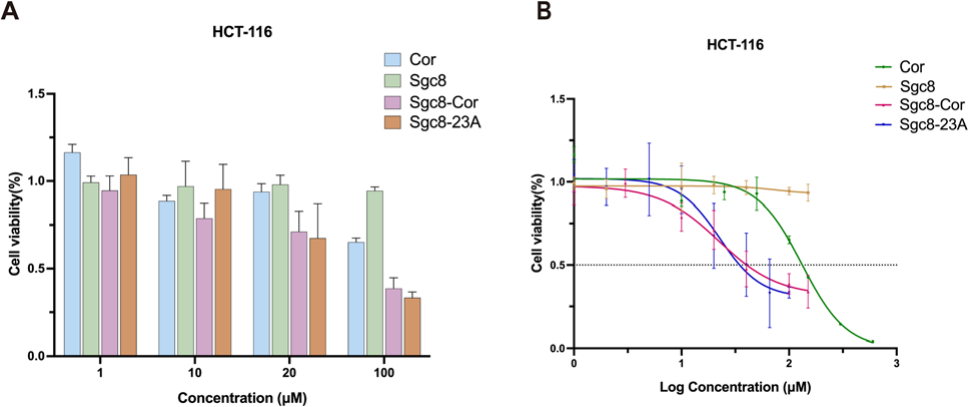
Tumor inhibitory activity of cordycepin-modified Sgc8c on HCT116 cells. (A) Cytotoxicity of free cordycepin (3′-dA), Sgc8c, Sgc8-Cor, and Sgc8-23A on HCT116 cells was evaluated using a CCK-8 assay across a concentration range of 1–100 μM (*n* = 3). (B) IC_50_ values of free 3′-dA, Sgc8c, Sgc8-Cor, and Sgc8-23A against HCT116 cells were determined by CCK-8 assay (*n* = 3). Error bars represent the standard error of the mean (SEM).

After confirming that Sgc8c modified with cordycepin retains strong targeting capability, we next examined its potential antitumor activity. Extensive studies indicated that Sgc8c exhibited minimal cytotoxicity toward tumor cells. By introducing cordycepin, we aimed to develop an Sgc8c derivative with both precise targeting and enhanced antitumor efficacy. We further evaluated the cytotoxic effects of Sgc8-23A on HCT116 cells using the CCK-8 assay, given that it had a *K*_d_ value closest to that of Sgc8c. As shown in Fig. 5A, Sgc8-23A exhibited significantly greater efficacy in inhibiting the growth of HCT116 cells compared to free 3′-dA across various concentrations. Additionally, the 48 h cytotoxicity test results, as presented in Fig. 5B, were used to compare 3′-dA, Sgc8c, Sgc8-23A and Sgc8-Cor. Notably, Sgc8-23A achieved an IC_50_ value of 22.65 μM, markedly lower than that of 3′-dA (IC_50_ = 131.4 μM), but comparable to that of Sgc8-Cor (IC_50_ = 22.19 μM). No substantial inhibition was observed in HCT116 cells treated with 100 μM Sgc8c, and this result was consistently validated across multiple experiments.

### *In vivo* antitumor evaluation of Sgc8-23A in a zebrafish PDX model

**Fig. 6:**
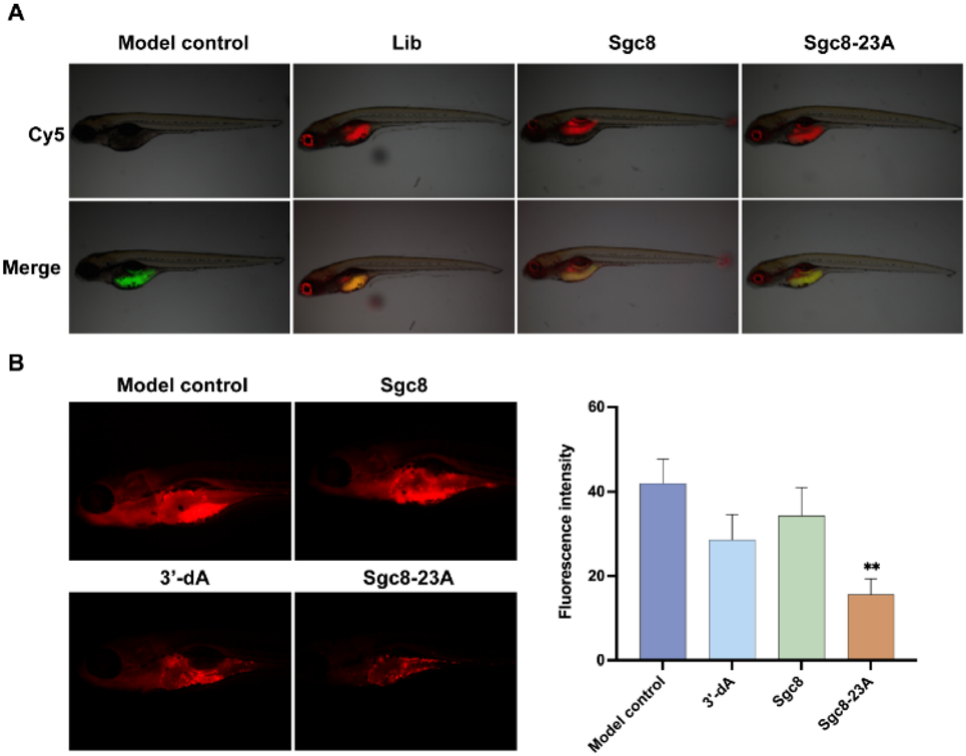
Antitumor activities in a patient-derived xenograft (PDX) model. (A) *In vivo* imaging of the HCT116 tumor xenograft zebrafish PDX model after Sgc8-23A, Sgc8c or Lib was injected intramuscularly. (B) Representative images of fluorescence intensity of tumor cells in the zebrafish PDX model under fluorescence microscopy at 48 h post-injection. Tumor cells were labeled with red fluorescence.

The zebrafish xenograft model, known for its ease of manipulation and rapid experimental turnaround, provided an ideal platform for our investigation of tumor progression.^32^ To assess the *in vivo* antitumor potential of Sgc8-23A, we established an optimized PDX model of colorectal cancer in zebrafish (*Danio rerio*). Cy5-labeled Sgc8-23A was administered intravenously at a dose of 10 ng per fish, along with a positive control (aptamer Sgc8c) and a negative control (Lib, random sequences). After injection, the zebrafish were incubated at 35 °C for 24 hours. Seven zebrafish were randomly chosen from each group (*n* = 7), and fluorescence microscopy was employed to assess the distribution of the samples. As illustrated in Fig. 6A, Sgc8-23A, like Sgc8c, exhibited preferential accumulation in the intestines, blood vessels, and tumor cells, underscoring its targeted affinity for tumor cells. Conversely, Lib was predominantly localized in the intestines and blood vessels. Then, to evaluate the antitumor potential of Sgc8-23A, we randomly divided the zebrafish into four groups, including control, Sgc8c, 3′-dA, and Sgc8-23A. Following 48 h of treatment, zebrafish from the experimental groups were randomly chosen for fluorescence microscopy analysis. As displayed in Fig. 6B, red fluorescence intensity in the Sgc8-23A group was significantly lower than that of the control group after exposure to the various compounds. Quantitative analysis using ImageJ software revealed a marked reduction in the overall tumor cell density after Sgc8-23A treatment. Notably, Sgc8-23A demonstrated significantly enhanced antitumor effects compared to 3′-dA. These findings underscore the promising therapeutic potential of Sgc8-23A.

## Conclusions

In summary, aptamer Sgc8c represents a promising tool for targeted cancer therapy and diagnostics. However, it faces several limitations, including insufficient antitumor activity, and inadequate stability and circulation time *in vivo*. In our work, dual-function cordycepin has been used to improve the antitumor efficacy and stability of Sgc8c through the substitution of adenine nucleotides. We have shown that cordycepin-modified Sgc8c, or Sgc8-23A, exhibits remarkable antitumor activity and specific targeting capabilities, opening new avenues in the field of targeted drug delivery. Our research has laid the groundwork for more efficient and adaptable strategies to enhance aptamer performance. We anticipate that this strategy will further evolve, providing innovative solutions for advancing targeted cancer treatment and diagnostic methods.

## Ethical statement

All zebrafish experiments were conducted in strict accordance with the guidelines for the care and use of laboratory animals and were approved by the Institutional Animal Care and Use Committee (IACUC) of Hangzhou Hunter Biotechnology Co., Ltd (IACUC approval number: IACUC-2024-9368-01).

## Author contributions

Fei Gao conducted the majority of the biological and cell-based assays. Na Li synthesized the target compounds, with additional contributions from Shuyue Fu in their synthesis. Jinsong Peng assisted with data analysis. Shipeng He, Ruowen Wang, and Weihong Tan conceptualized the project, provided overall guidance, contributed to data analysis, and participated in the writing, review, and editing of the manuscript. All authors have read and approved the final manuscript.

## Conflicts of interest

There are no conflicts to declare.

## Supplementary Material

SC-016-D5SC02571K-s001

## Data Availability

The datasets supporting this article have been uploaded as part of the ESI† material.
