# Supplementary material for: Cordycepin: a dual-function molecular element for aptamer engineering with enhanced anticancer activity
Source: Chem Sci. 2025 Jun 30;16(29):13235–40. doi: 10.1039/d5sc02571k (PMC12208130; doi:10.1039/d5sc02571k)
Supplement: SC-016-D5SC02571K-s001 [file SC-016-D5SC02571K-s001.pdf]

Supplementary Information (SI) for Chemical Science.
This journal is © The Royal Society of Chemistry 2025

SUPPLEMENTARY DATA

Cordycepin: a bifunctional molecular element for aptamer functionalization

Fei Gao[a,#], Na Li[a,#], Shuyue Fu[a,#], Jinsong Peng[a], Shipeng He[a,*], Ruowen Wang[b,*],Weihong Tan[b,*]

[a]Institute of Translation Medicine, School of Life Science, Shanghai University, Shanghai, 200444, China.

[b]Institute of Molecular Medicine (IMM), Renji Hospital, State Key Laboratory of Systems Medicine for Cancer, Shanghai Jiao Tong University School of Medicine, and College of Chemistry and Chemical Engineering, Shanghai Jiao Tong University, Shanghai 200240, China.

Table of Contents

1. General information

2. Synthesis of Cordycepin Phosphoramidite.

3. Copies of HPLC and Mass Spectra

4. Copies of NMR Spetra

1. General information

General chemicals were purchased from Tansoole (Shanghai) Co., Ltd., J&K Scientific Ltd., and Sigma-Aldrich. Cordycepin was obtained from Bokachem (Shanghai, China). RPMI 1640 medium, fetal bovine serum (FBS), and Dulbecco's phosphate-buffered saline (DPBS) were acquired from Thermo Fisher Scientific (USA). DMSO, MTT solution and Hoechst 33342 were purchased from Beyotime (Shanghai, China). All other reagents were sourced from domestic suppliers and used as received. All reagents were handled according to the manufacturers' instructions to ensure proper storage and experimental conditions.

All oligonucleotide sequences are listed in the table below. Cordycepin Phosphoramidite are denoted as **A'** to replace Adenine in the below sequences.

Table S1. Detailed sequence data for cordycepin-modified Sgc8 and Lib

DNA	Sequence (5'-3')
Sgc8-Cy5	ATCTAACTGCTGCGCCGCCGGGAAAATACTGTACGGTTAGA-Cy5
Sgc8-1A-Cy5	**(A')**TCTAACTGCTGCGCCGCCGGGAAAATACTGTACGGTTAGA-Cy5
Sgc8-6A-Cy5	ATCTA(**A'**)CTGCTGCGCCGCCGGGAAAATACTGTACGGTTAGA-Cy5
Sgc8-23A-Cy5	ATCTAACTGCTGCGCCGCCGGG(**A'**)AAATACTGTACGGTTAGA-Cy5
Sgc8-28A-Cy5	ATCTAACTGCTGCGCCGCCGGGAAAAT(**A'**)CTGTACGGTTAGA-Cy5
Sgc8-33A-Cy5	ATCTAACTGCTGCGCCGCCGGGAAAATACTGT(**A'**)CGGTTAGA-Cy5
Sgc8-Cor	ATCTAACTGCTGCGCCGCCGGGAAAATACTGTACGGTTAGA(**3'dA**)- Cy5
Lib-Sgc8-Cy5	ATCTAACTGATTATTATTATTATTATTATTATTCGGTTAGA-Cy5

[a] All DNA probes were synthesized by Sangon Biotech CO., Ltd;

Cell Culture. HCT116 and Ramos cell lines were obtained from the American Type Culture Collection (ATCC, Manassas, VA, USA). Both cell lines were maintained in RPMI-1640 medium supplemented with 10% fetal bovine serum (FBS) and 1% penicillin-streptomycin. Cells were incubated at 37 °C in a humidified atmosphere with 5% CO_2.

Buffers. Washing buffer was prepared by adding 2.25 g and 2.5 mL $MgCl_2$ (1 M) into 500 mL DPBS. Binding buffer was prepared by adding 50 mg yeast tRNA and 500 mg BSA into 500 mL washing buffer.

Flow Cytometric Analysis. To evaluate the binding affinity of aptamers, HCT116 cells were initially treated with Accutase™ Cell Detachment Solution, followed by three washes. Cells (2 × 10^5) were incubated with 250 nM of Cy5-labeled sgc8 or its variants (sgc8-1A, sgc8-6A, sgc8-23A, sgc8-28A, sgc8-33A) in 200 μL of binding buffer at 4 °C for 30 min in the dark. After incubation, cells were resuspended in 200 μL of washing buffer and analyzed using a Beckman flow cytometer to measure fluorescence intensity. For determining the dissociation constant (Kd) of the aptamer–cell interactions, the same flow cytometry protocol was followed using varying concentrations of each aptamer. Kd values were obtained by fitting fluorescence versus aptamer concentration data using GraphPad Prism software.

Confocal imaging. HCT116 cells were seeded in glass-bottom dishes (NEST Biotechnology, Jiangsu, China) at a density of 3 × 10^5 cells per well and incubated overnight. Cells were then treated with 500 nM Cy5-labeled sgc8 or its variants (sgc8-1A, sgc8-6A, sgc8-23A, sgc8-28A, sgc8-33A) at 37 °C for 2 h. After incubation, unbound aptamers were removed by washing three times with washing buffer. Hoechst 33342 was applied to stain the nuclei, and cells were washed twice. Subsequently, cells were incubated in medium containing 10% FBS at 37 °C for 1 h. Prepared samples were then visualized using a Leica TCS SP8 confocal microscope.

Circular dichroism measurements. The CD spectra were recorded on a Jasco-1500 spectropolarimeter. Samples of sgc8 and its variants (sgc8-1A, sgc8-6A, sgc8-23A, sgc8-28A, sgc8-33A) were prepared in DPBS supplemented with 5 mM $MgCl_2$ to reach a final concentration of 10 μM in a total volume of 200 μL. Measurements were conducted at room temperature using a 1 mm path length quartz cuvette. Spectra were collected by averaging three scans from 300 to 200 nm, with a data pitch of 0.5 nm, bandwidth of 2 nm, and a scan speed of 50 nm/min.

Stability analysis of aptamers. For stability assays, 1 μM Cy5-labeled sgc8 and its variants (sgc8-1A, sgc8-6A, sgc8-23A, sgc8-28A, sgc8-33A) were incubated in RPMI 1640 medium supplemented with 10% fetal bovine serum (FBS) at 37 °C. At specified intervals (0, 2, 4, 6, 8, 10, 24, 36, and 48 h), samples were removed from the incubator, heated to 95 °C for 10 min to inactivate enzymes, and stored at −20 °C until further analysis. For PAGE analysis, 10 μL of each DNA sample was combined with 2 μL of 6× loading buffer (Sangon) and loaded onto a 10% polyacrylamide gel

prepared with electrophoresis buffer (9 mM Tris, 9 mM boric acid, and 1 mM EDTA, pH 8.0). After electrophoresis, gels were imaged using a molecular imager (GE Healthcare).

Molecular docking. The DNA structure was predicted using the client-provided sequence through AlphaFold3. Subsequently, the 23rd nucleotide was mutated in PyMOL. The PTK7 protein structure was obtained from the open-access online database UniProt (Q13308). Protein protonation was performed under neutral conditions (pH = 7) using the H++ 3.0 online server, followed by charge assignment based on the Amber14SB force field via UCSF Chimera. Molecular docking was carried out using the HDOCK server, which employs the empirical iterative scoring function ITScorePP to model and assess protein-protein interactions. A maximum of 100 docking poses were generated, with the top 10 poses selected for further analysis. The docking scores, which were negative, indicated binding affinity, with more negative values corresponding to stronger binding interactions. The highest-ranking pose was selected for subsequent analysis. 3D visualization of the selected structure was performed using PyMOL 2.5.7, and ligand-protein interaction diagrams were generated using LigPlot+ 2.1.

Cell Viability Assay. For cell viability analysis, HCT116 cells were seeded in 96-well plates at a density of approximately 5000 cells per well and incubated overnight to allow for adherence. Cells were then treated with aptamers at various concentrations (1–100 μM) in FBS-free medium for 48 h at 37 °C. Following treatment, 10 μL of CCK-8 solution was added to each well, and cells were incubated for an additional 2 h at 37 °C. The absorbance at 450 nm was measured using a BioTek Synergy H1 microplate reader to assess cell viability.

Animal Care and Husbandry. All zebrafish experiments were conducted in strict accordance with the guidelines for the care and use of laboratory animals and were approved by the Institutional Animal Care and Use Committee (IACUC) of Hangzhou Hunter Biotechnology Co., Ltd. (IACUC approval number: IACUC-2024-9368-01). Zebrafish (wild-type AB strain) were maintained in aquaria at 28 °C with standardized fish water (composition: 200 mg of instant sea salt per liter of reverse osmosis water, conductivity 450–550 μS/cm, pH 6.5–8.5, hardness 50–100 mg/L $CaCO_3$). Zebrafish used in this study were bred by natural pair mating and supplied by our institution's Aquatic Center under animal use permit no. SYXK (Zhe) 2022-0004. All procedures adhered to

AAALAC International accreditation standards (certification no. 001458) and were reviewed under IACUC protocol no. IACUC-2024-4071-01. Embryos at 2 days postfertilization (2 dpf) were selected for tumor efficacy assays. Colorectal tumor tissue (sample no. CRC2023072001) was obtained from Shandong Provincial Hospital.

Xenograft Zebrafish Model and Sample Distribution. To establish a colorectal cancer xenograft model, HCT116 cells were labeled with green fluorescent DiO dye (Thermo Fisher Scientific, Waltham, MA) and microinjected into the yolk sac of wild-type AB strain zebrafish embryos at 2 days post-fertilization (dpf). Each zebrafish received approximately 200 cells, with an effective dose of 10 ng/fish. Following injection, xenografted zebrafish larvae were maintained at 35 °C. At 3 dpf, larvae with consistent tumor cell localization were selected under a fluorescence microscope and randomly assigned to six-well plates, with 30 fish per well for each experimental group. Samples were administered directly into the yolk sac, and a model control group was established. Zebrafish were incubated at 35 °C and imaged at 24 hours post-treatment; at each time point, seven fish per group were photographed to evaluate sample distribution using fluorescence microscopy.

Evaluation of Antitumor Efficacy in Zebrafish PDX Model. Tumor tissue was washed in PBS, cut into small fragments, and subjected to enzymatic digestion in a mixed enzyme solution (collagenase, hyaluronidase, and DNase I). After 5 minutes of hand-shaken incubation at 37 °C, digestion was stopped by transferring the supernatant to DMEM with 10% FBS. The cell suspension was filtered through a 70 μm mesh, centrifuged, and washed with PBS. Cells were labeled with CM-DiI (4 μL/mL) and microinjected (200 cells per embryo) into the yolk sac of wild-type AB strain zebrafish embryos at 2 days post-fertilization (dpf). At 3 dpf, zebrafish with consistent tumor cell labeling were randomly assigned to 6-well plates, with 20 fish per well. Treatments, including sgc8, sgc8-23A or 3'-dA (4 ng/fish), were administered via intravenous injection. Mortality was monitored daily, and deceased specimens were promptly removed. At 5 dpf, tumor growth was assessed by imaging ten fish per group under fluorescence microscopy.

2. Synthesis of Cordycepin Phosphoramidite.

Preparation of Compound 1

Cordycepin (1.5004 g, 5.97 mmol) was placed in a 100 mL dry eggplant flask, evacuated, and backfilled with argon (Ar). It was dissolved in 40 mL of anhydrous pyridine and stirred until a homogeneous solution was obtained. Under an ice bath, TMSCl (3.8 mL) was added, and the reaction was stirred at room temperature for 30 min. Subsequently, BzCl (2.1 mL) was added under ice bath conditions, and the reaction was allowed to proceed at room temperature for 3 h. The reaction was quenched and deprotected by adding 30 mL of a saturated sodium bicarbonate solution, and stirring was continued for an additional 30 min. Thin-layer chromatography (TLC) confirmed the completion of the reaction. The solvent was removed under reduced pressure to yield a crude product, which was purified by silica gel column chromatography (dichloromethane = 10:1) to give Compound **1** as a white solid (0.8675 g, 2.44 mmol, 40.8% yield). **^{1}H NMR (400 MHz, $CDCl_3$)** δ 8.57 (s, 1H), 8.43 (s, 1H), 7.82 (d, J = 7.7 Hz, 4H), 7.47 (t, J = 7.5 Hz, 2H), 7.33 (t, J = 7.6 Hz, 4H), 5.85 (d, J = 3.6 Hz, 1H), 4.72 (m,1H), 4.45 (t, J = 7.1 Hz, 1H), 3.91 (d, J = 12.6 Hz, 1H), 3.53 (d, J = 12.7 Hz, 1H), 2.33 (m,1H), 2.00 (dt, J = 12.8, 6.2 Hz, 1H). **^{13}C NMR (101 MHz, $CDCl_3$)** δ 172.4, 151.9, 151.8, 151.6, 144.6, 133.7, 133.2, 129.92, 129.5, 128.8, 128.3, 128.1, 93.1, 81.1, 75.0, 63.5, 33.2.

Preparation of Compound 2

Compound **1** (0.7340 g, 2.07 mmol) was placed in a 50 mL eggplant flask, evacuated, and backfilled with argon (Ar). Under argon atmosphere, 10 mL of anhydrous pyridine was added via syringe, and the solution was stirred until homogeneous. DMTrCl (0.8399 g) was then added, and the reaction was allowed to proceed overnight at room temperature. The reaction was quenched by adding 5 mL of methanol, and the solvent was removed under reduced pressure to yield a crude product. The crude product was purified by silica gel column chromatography (petroleum ether:ethyl acetate = 1:1) to afford Compound **2** as a white solid (0.6873 g, 1.04 mmol, 50.6% yield). **^{1}H NMR (400 MHz, DMSO-H6)** δ 8.75 (m, 2H), 7.71 (t, J = 7.7 Hz, 3H), 7.53 (d, J = 7.5 Hz, 2H), 7.36 (m, 5H), 7.17 (m, J = 7.5 Hz, 6H), 6.80 (t, J = 8.9Hz, 3H), 6.07 (s, 1H), 5.80 (s, 1H), 4.03 (s, J = 7.1 Hz, 1H), 3.68 (m, J = 3.0 Hz, 5H), 3.25 (d, 2H), 1.99 (d, 2H), 1.25 (d, 2H).**^{13}C NMR (101 MHz, $CDCl_3$)** δ 172.3, 158.7, 158.6, 151.8, 147.4, 144.5, 144.4, 143.2, 139.5, 135.9, 135.8, 135.7, 134.1, 134.0, 133.2, 133.1, 133.0, 130.2, 130.1, 130.0, 129.5, 129.0, 129.0, 128.8, 128.1, 128.1, 128.0, 127.96 127.9, 127.8, 113.4, 93.4, 93.1, 77.3, 76.1, 64.9, 55.3, 34.4.

Preparation of Compound 3

Compound **2** (0.6870 g, 1.04 mmol) was placed in a 50 mL dry eggplant flask, evacuated, and backfilled with argon (Ar). Anhydrous DCM (10 mL) was added, and the solution was stirred until homogeneous. DIPEA (0.82 mL) was added, followed by the addition of CED-Cl (0.90 mL) under ice-bath cooling. The reaction was stirred at room temperature for 1 h, and TLC confirmed the reaction's completion. The mixture was then extracted twice with DCM and saturated solutions of sodium bicarbonate and sodium chloride. The organic phase was collected, dried over anhydrous sodium sulfate, filtered, and concentrated under reduced pressure. The residue was mixed with silica gel and purified by silica gel column chromatography (petroleum ether:ethyl acetate = 1:1) to yield Compound **3** as a white solid (0.8181 g, 0.95 mmol, 91.2% yield). **^{1}H NMR (400 MHz, $CDCl_3$)** δ 8.57 (s, 1H), 8.29 (s, 1H), 7.80 (m, J = 7.6 Hz, 4H), 7.45 (m, 4H), 7.15 (d, J = 7.2 Hz, 2H), 6.76 (m, J = 8.3 Hz, 4H), 6.26 (m, 5H), 4.97 (s, 1H), 4.63 (s, 1H), 3.82 (s, 1H), 3.69 (s, 1H), 3.61 (d, 2H), 3.29 (s, 1H), 2.57 (s, 1H), 1.15 (s, 1H).**^{31}P NMR (162 MHz, CDCl3)** δ 150.21, 149.27.

3. Copies of HPLC and Mass Spectra.

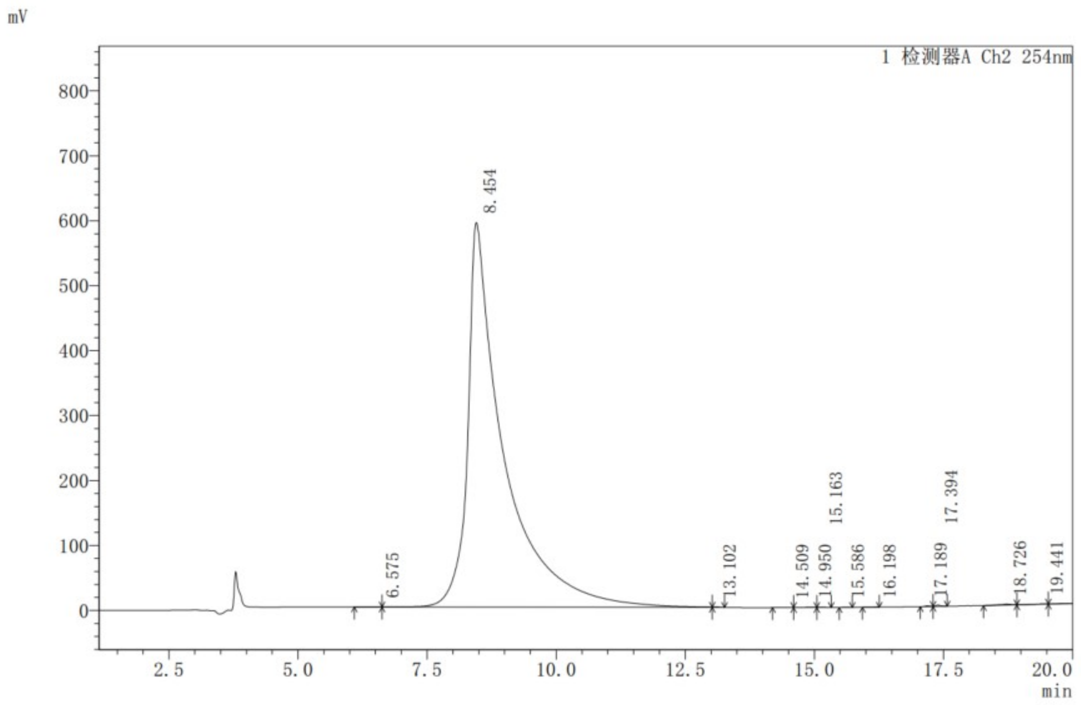

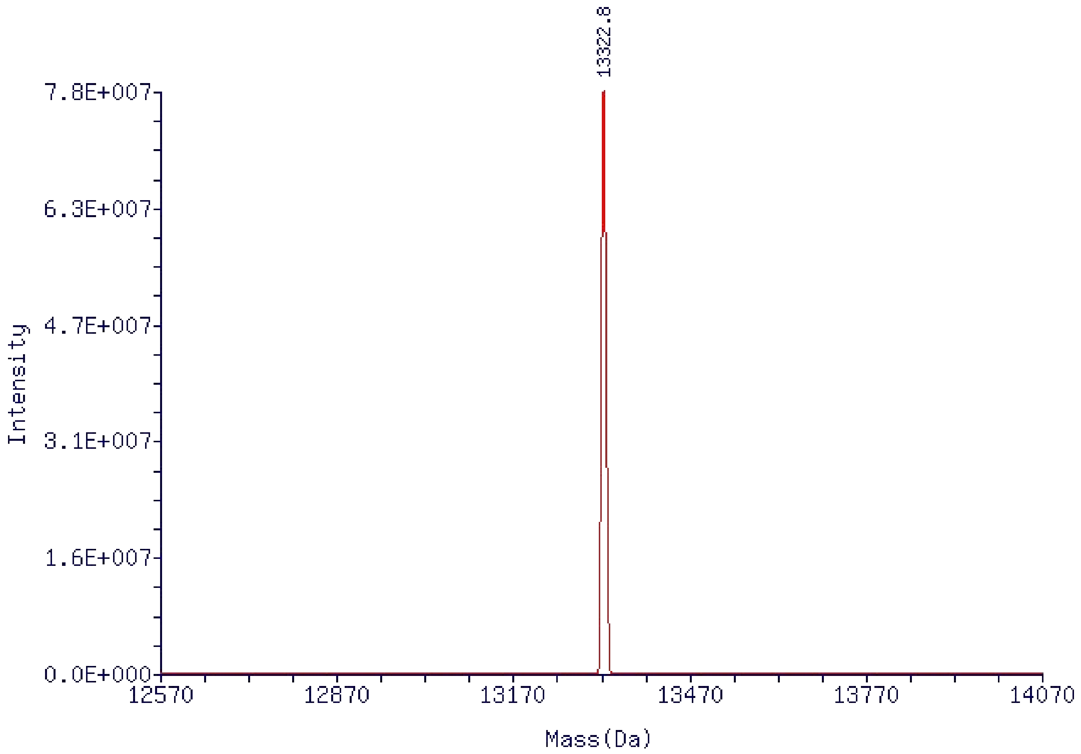

Figure S1. (A) HPLC of Sgc8c; (B) ESI-MS analysis of Sgc8c by Sangon (Shanghai). Calculated molecular weight was 13323.05, and observed DNA peak was 13322.8.

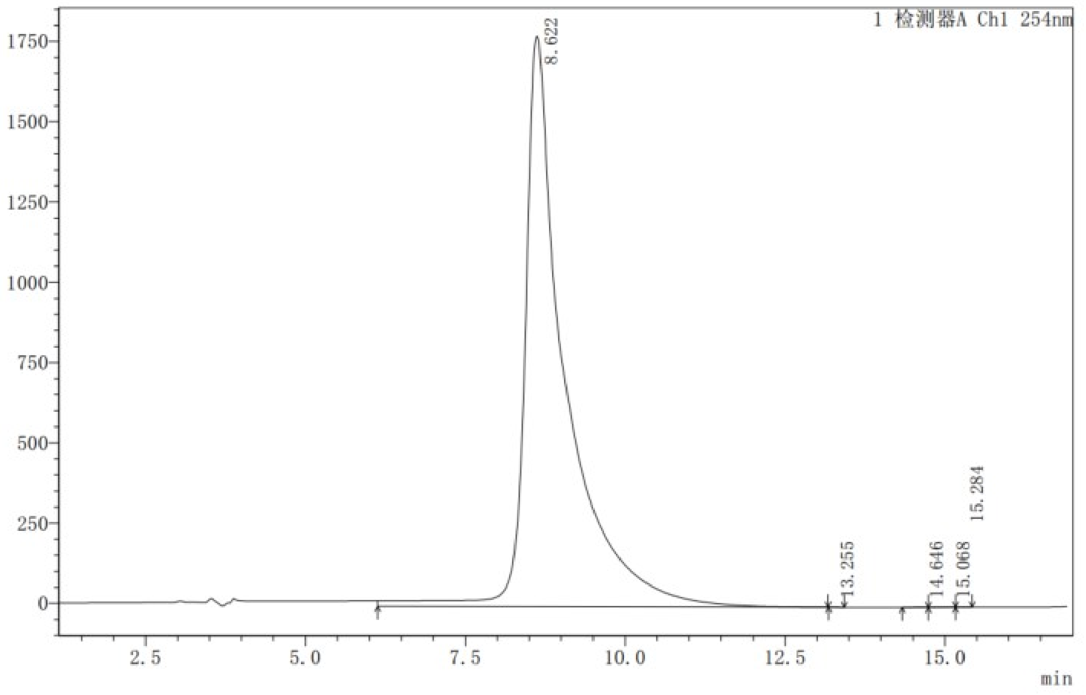

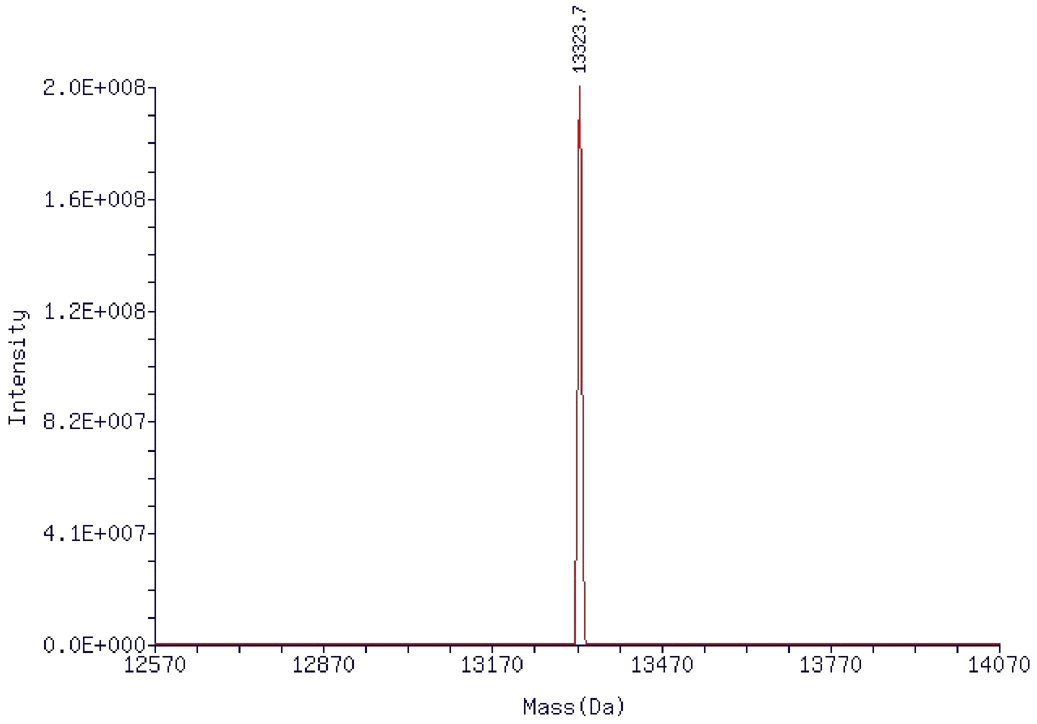

Figure S2. (A) HPLC of Sgc8-1A; (B) ESI-MS analysis of Sgc8-1A by Sangon (Shanghai). The calculated molecular weight was 13323.05, and the observed DNA peak was 13323.7.

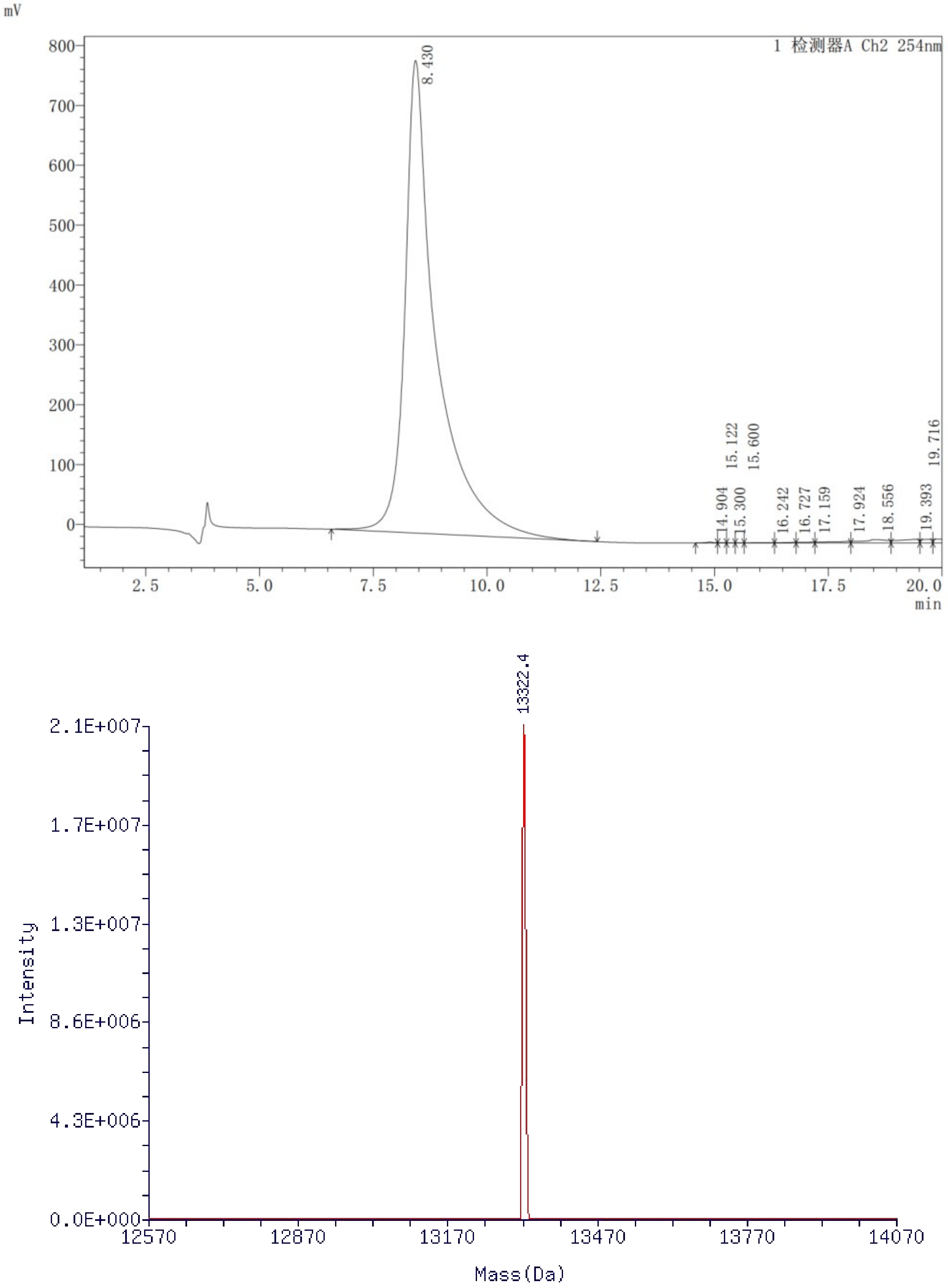

Figure S3. (A) HPLC of Sgc8-6A; (B) ESI-MS analysis of Sgc8-6A by Sangon (Shanghai). The calculated molecular weight was 13323.05, and the observed DNA peak was 13322.4.

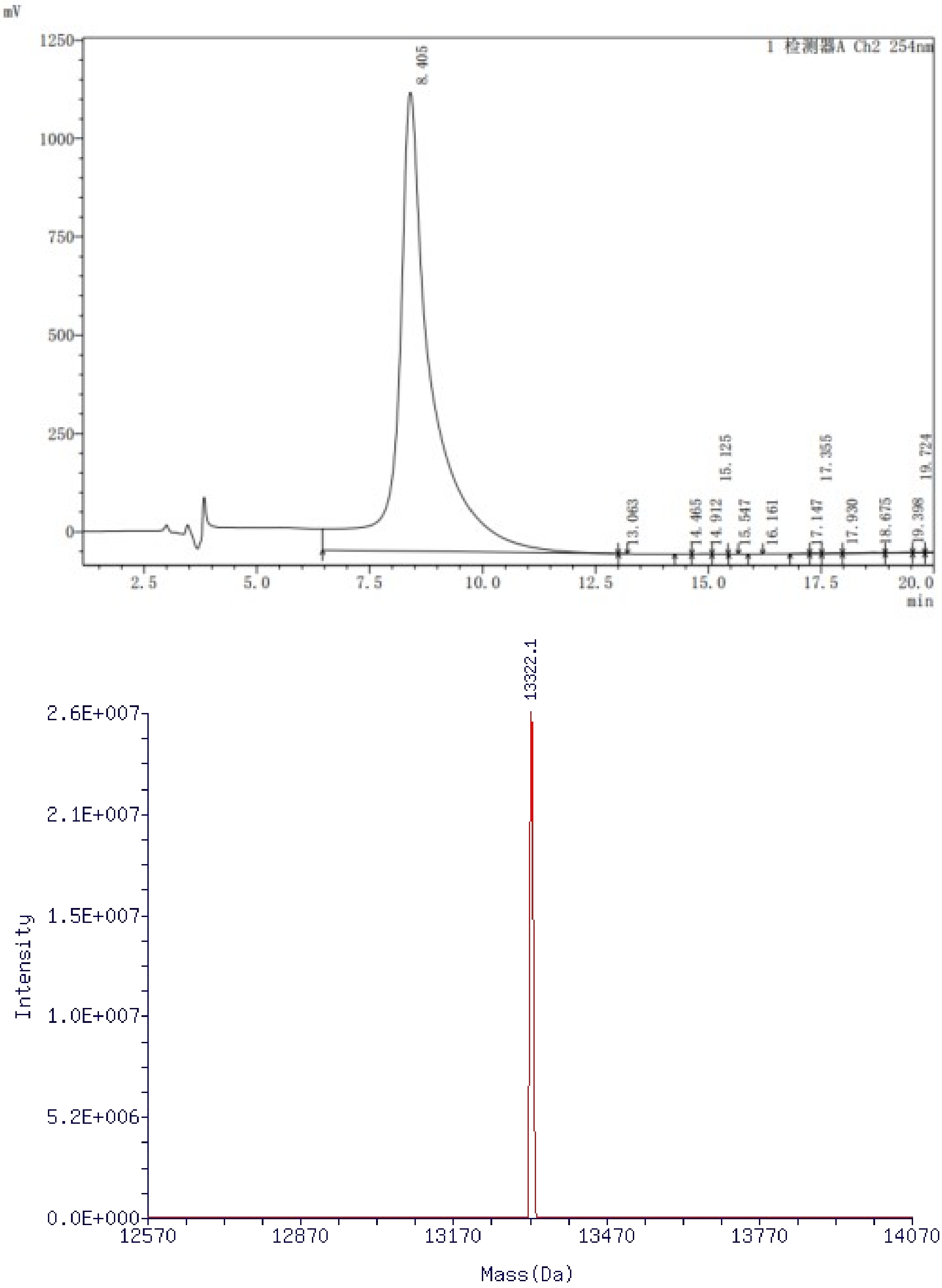

Figure S4. (A) HPLC of Sgc8-23A; (B) ESI-MS analysis of Sgc8-23A by Sangon (Shanghai). The calculated molecular weight was 13323.05, and the observed DNA peak was 13322.1.

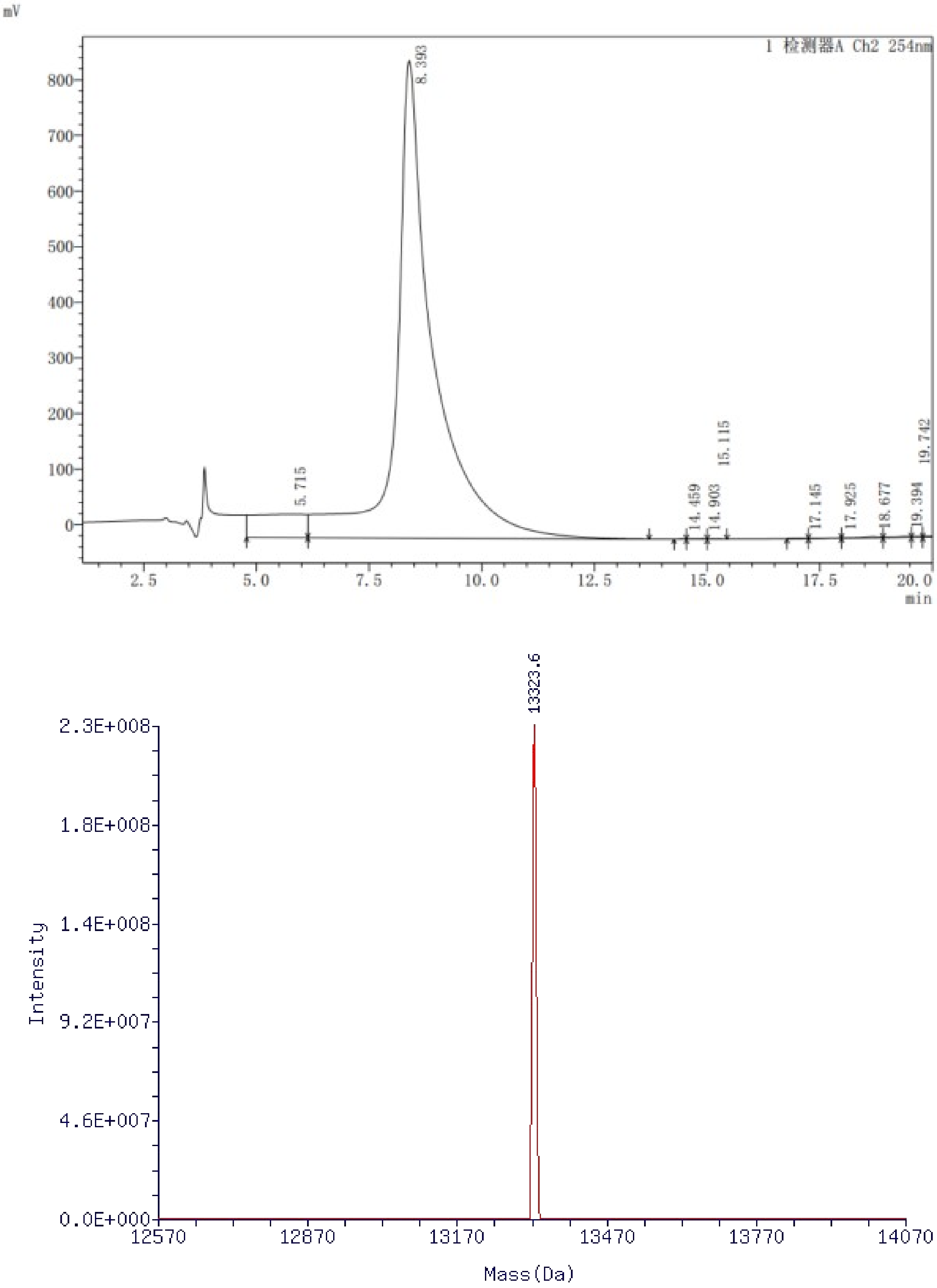

Figure S4. (A) HPLC of Sgc8-28A; (B) ESI-MS analysis of Sgc8-28A by Sangon (Shanghai). The calculated molecular weight was 13323.05, and the observed DNA peak was 13323.6.

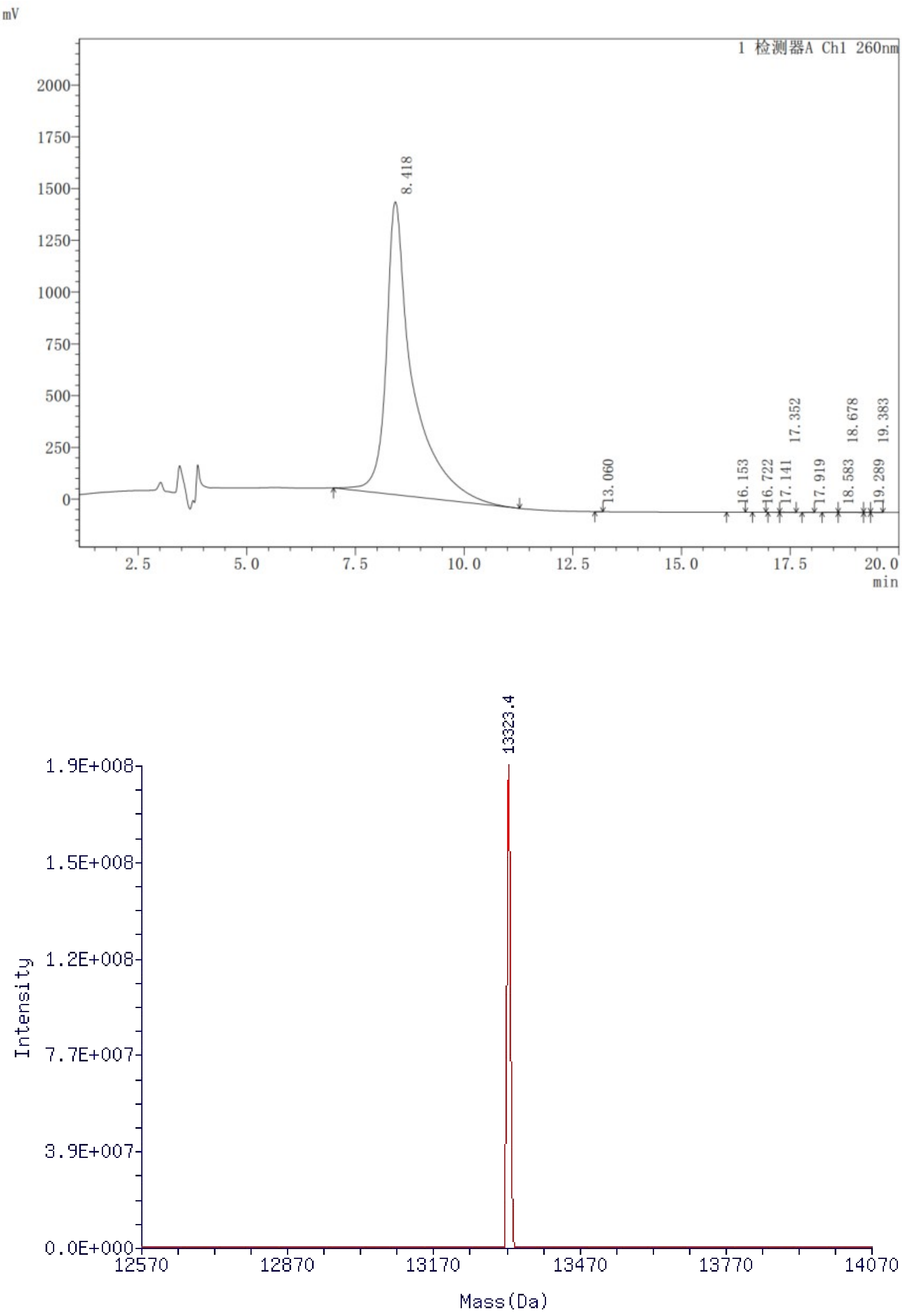

Figure S5. (A) HPLC of Sgc8-33A; (B) ESI-MS analysis of Sgc8-33A by Sangon (Shanghai). The calculated molecular weight was 13323.05, and the observed DNA peak was 13323.4.

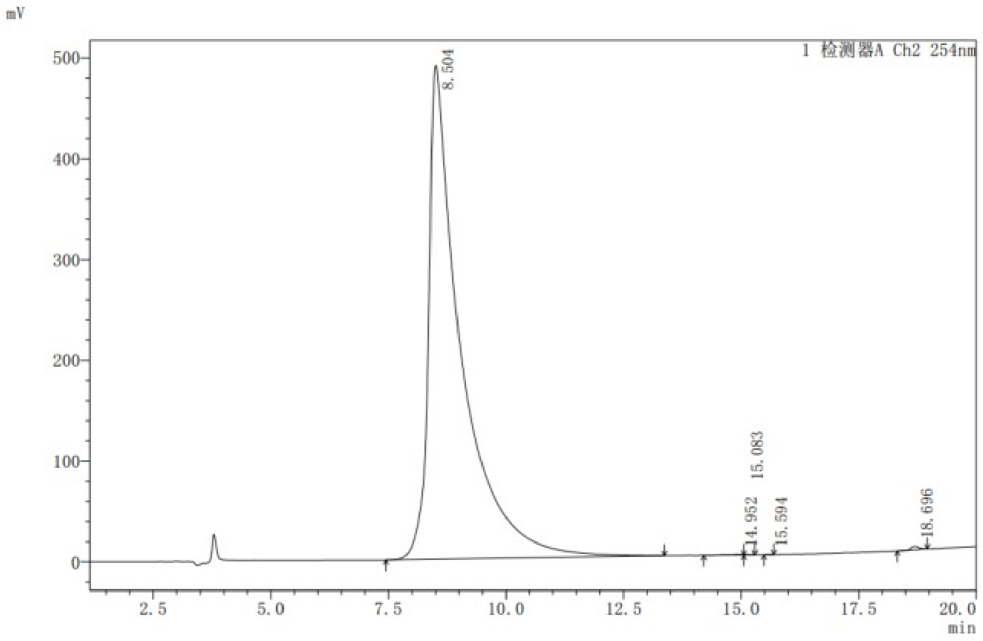

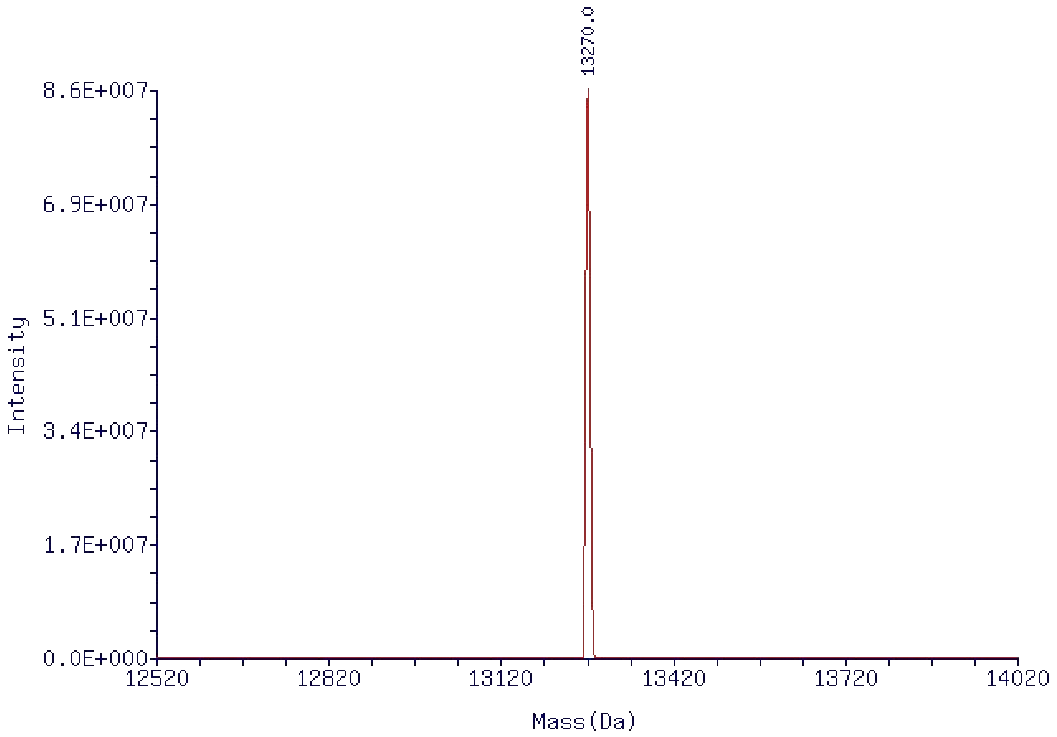

Figure S6. (A) HPLC of Lib; (B) ESI-MS analysis of Lib by Sangon (Shanghai). The calculated molecular weight was 13271.08, and the observed DNA peak was 132370.0.

4. Copies of NMR spectra

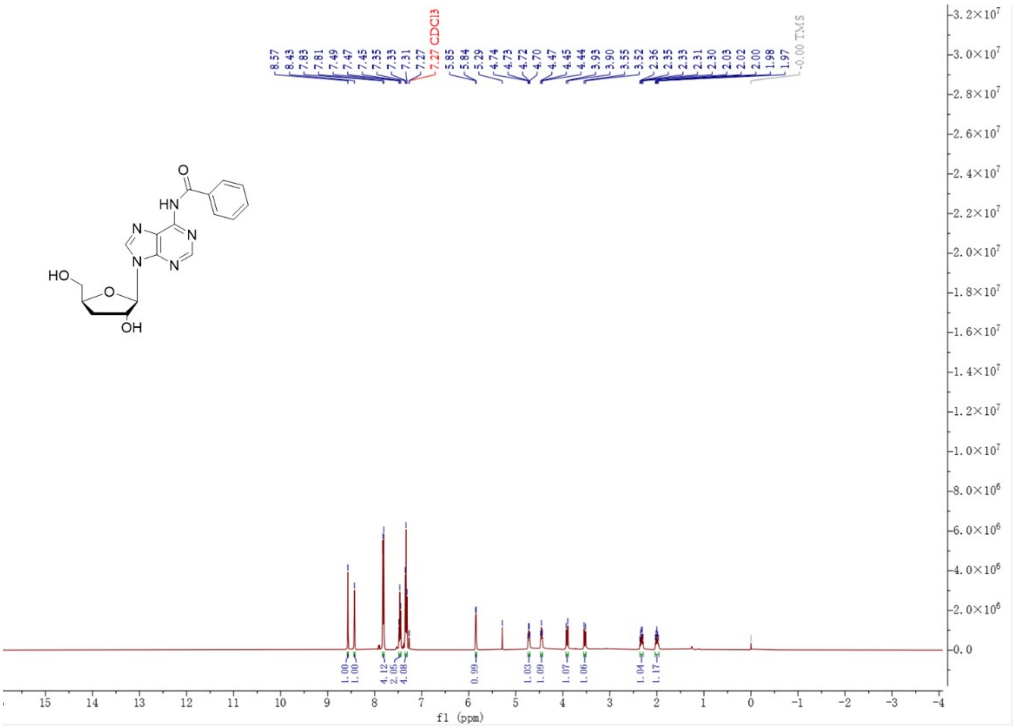

Figure S7. ^{1}HNMR of Compound **1**

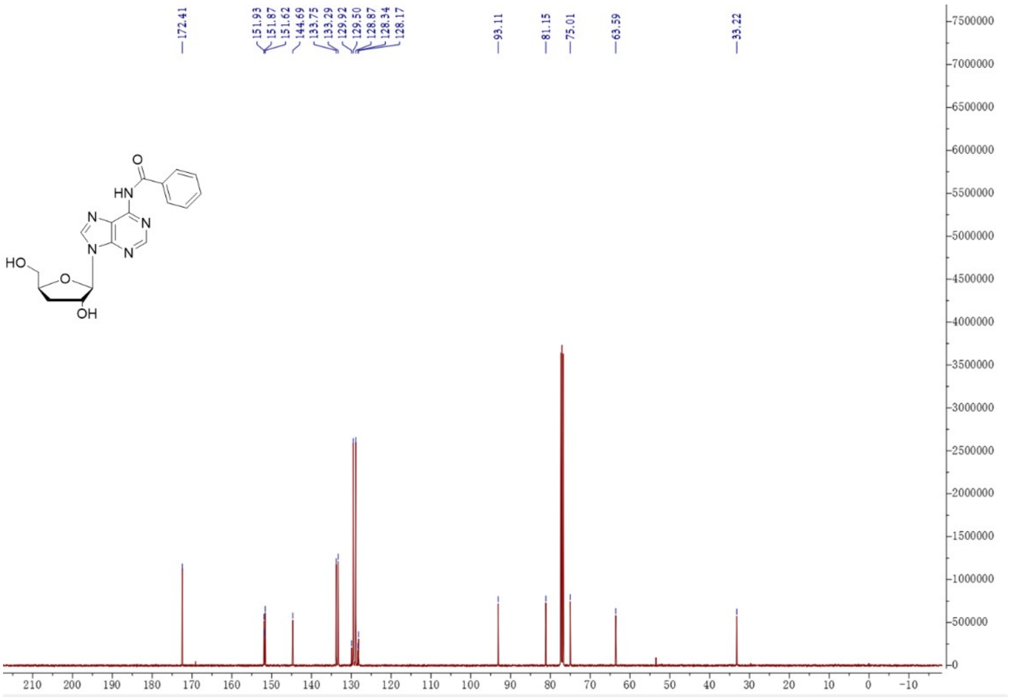

Figure S8. ^{13}CNMR of Compound **1**

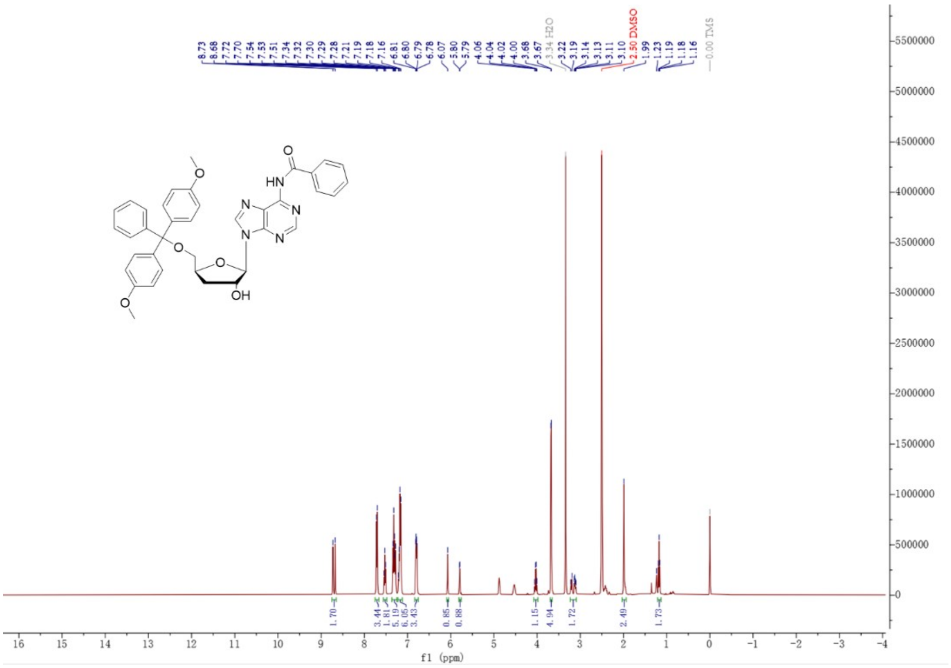

Figure S9. ^{1}HNMR of Compound **2**

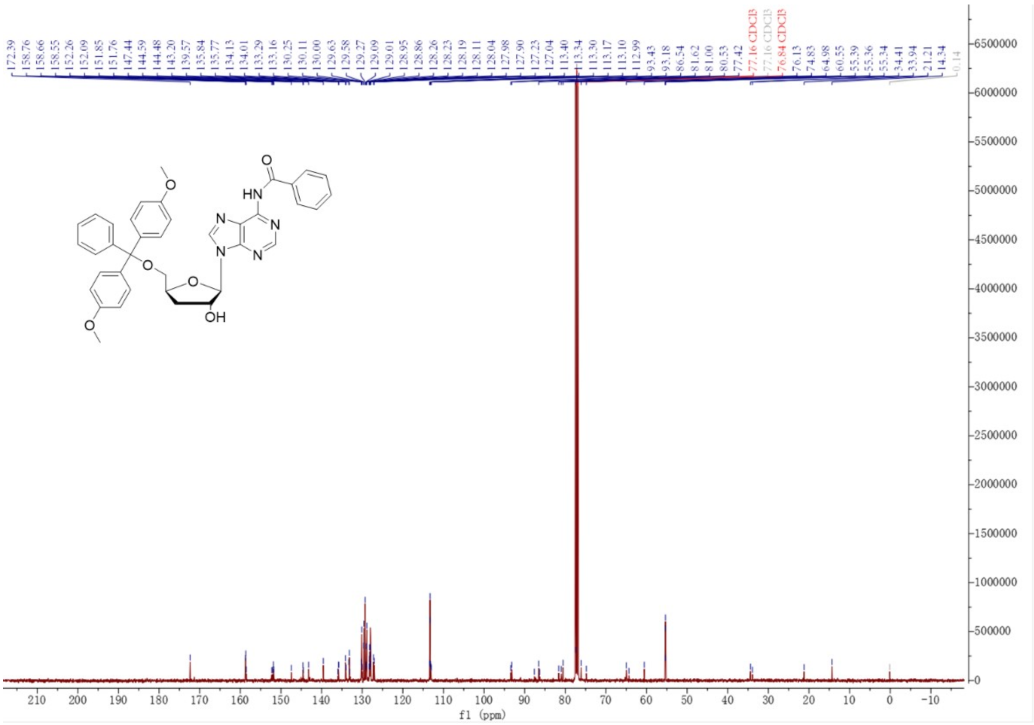

Figure S10. ^{13}CNMR of Compound **2**

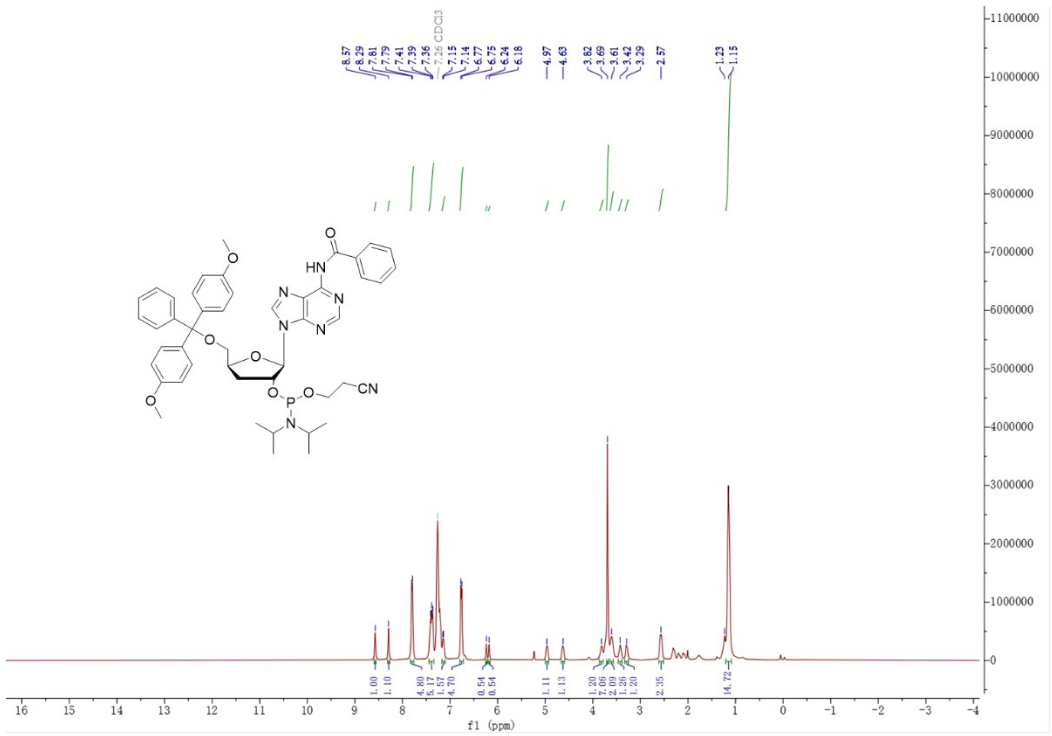

Figure S10. ^{1}HNMR of Compound **3**

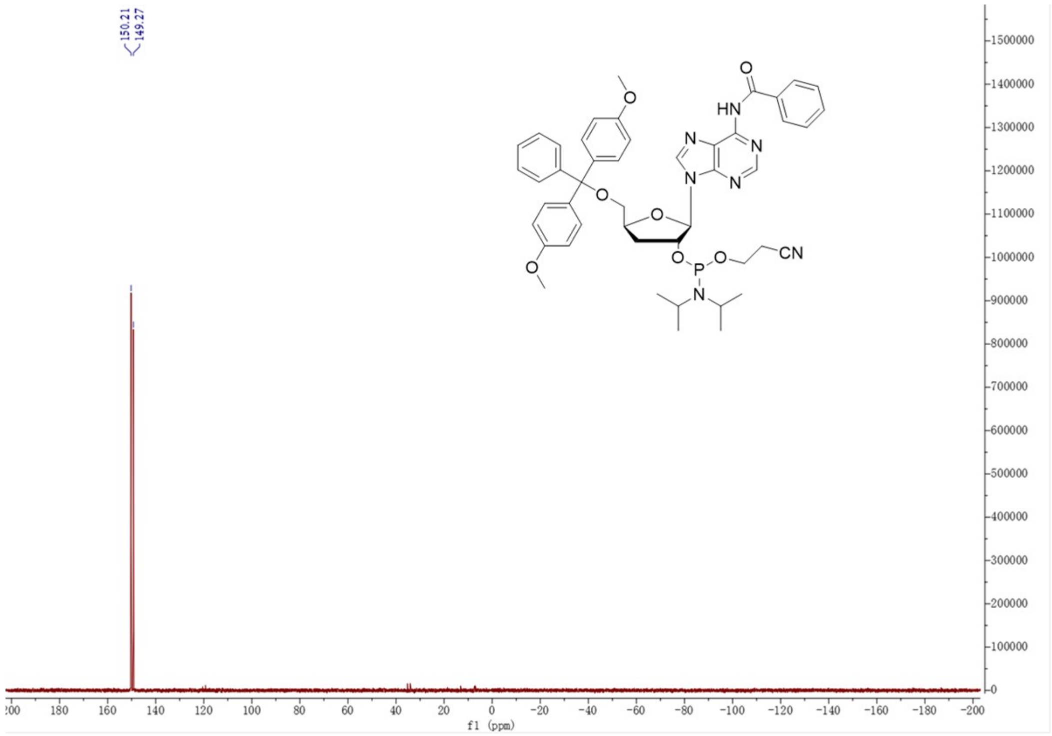

Figure S11. ^{31}PNMR of Compound **3**
